# Psychological risk factors for a first hamstring strain injury in soccer: a qualitative study

**DOI:** 10.3389/fspor.2024.1377045

**Published:** 2024-06-14

**Authors:** Diane Baize, Fabienne d’Arripe-Longueville, Enzo Piponnier, Stéphanie Scoffier-Meriaux

**Affiliations:** Laboratoire Motricité Humaine Expertise Sport Santé (LAMHESS, UPR 6312), Sport Science, Université Côte d’Azur, Nice, France

**Keywords:** sports injury model, athletic identity, football, primary prevention, athletic injuries, health literacy

## Abstract

**Introduction:**

Hamstring strain injuries (HSIs) remain one of the most burdensome injuries in soccer. Current recommendations to prevent sports injuries suggest the importance for coaches and medical staff to consider psychological and contextual risk factors and to specify them according to the injury type and context. HSI risk factors in soccer have been widely investigated, mainly from physiological and biomechanical perspectives. However, psychological and health-related risk factors are still unexplored. Therefore, the objective of this study was to determine the psychological and health-related risk factors for a first HSI in male competitive soccer.

**Method:**

Individual semi-structured interviews were conducted with ten male competitive soccer players, who had recently sustained a HSI. Based on multifactorial models of sports injury causation, soccer players' individual, contextual, and situational risk factors at the time of their first HSI were investigated. Interviews were analyzed using thematic analysis with deductive and inductive approaches.

**Results:**

Individual psychological risk factors included common at-risk personality traits, obsessive passion for soccer with competitive motivational goals, strong athletic identity, and poor health literacy. The injured players were exposed to a controlling coaching style, with a fear of negative staff evaluations, and had recently experienced life stressors. They were injured during matches or overload periods and were highly engaged in the activity.

**Discussion:**

Previously injured soccer players exhibit a lack of perspective concerning the repercussions of their actions on their health. From a preventive viewpoint, these results suggest enhancing the players' health literacy, supporting their autonomy, and moderating the controlling coaching style.

## 1 Introduction

In male professional soccer players, hamstring strain injuries (HSIs) represented a quarter of all injuries (24%) in the 2021–22 season and were the cause of 20% of players' absences from training or competition (1). This is all the more concerning as HSI have a very high reinjury rate, ranging between 16% and 26% (1–3). They impact both amateur and professional soccer players (1, 4), with heavy costs for the individual players [e.g., psychological, functional, and career impacts (5)], as well as for the soccer clubs and society. From a preventive viewpoint, extrinsic and intrinsic HSI risk factors have been widely investigated, mainly from physiological and biomechanical perspectives (6, 7). Age and prior HSI are the most important risk factors, but they are not modifiable. Thus, HSI prevention targets risk factors that are physiological (e.g., low hamstring eccentric strength) or related to training load (e.g., spikes in acute training load) (8). However, this is insufficient since the incidence of HSIs has continued to rise since 2014 (1).

This divergence between research into risk factors and the effectiveness of prevention could be due to the reductive approach to risk factors. HSI risk factors have mostly been identified through protocols focusing on the independent effect of a risk factor on injury outcome (e.g., hamstring strength or flexibility), without considering the contextual determinants such as the training load (9). As sports injuries are multifactorial (6), recent studies recommend including psychosocial risk factors in the development of preventive sequences and considering risk factors as a contextually driven web of determinants (e.g., sport, structure of practice) (9). Indeed, the role of psychological risk factors in sports injuries is well-established (10–12), and neglecting them may lead to incomplete or inefficient injury prevention efforts, including over the long term. Psychological factors, encompassing risk perception, decision-making, stress management, self-regulation, communication, and education, profoundly influence injury prevention and risk awareness in sports like soccer. By addressing these factors through targeted interventions, athletes can enhance their ability to identify and mitigate risks, ultimately reducing the incidence injuries. However, psychological factors have not been documented for HSI; they are occasionally referred to as “psychosocial factors” without specific elaboration (13). The only investigation that focused on athletes' intentions at the moment of injury (14) revealed that athletes disregarded their pain sensations and aimed to surpass their limits when they were injured. In a soccer context, quantitative studies confirmed that the leadership style of the head coach (15) and stress-injury model determinants (10, 16) (i.e., personality, history of stressors, coping strategies) are injury risk factors. However, goalkeeper concussions and striker HSIs follow different paths. To face the difficult problem of HSIs in soccer, we must understand the occurrence of this specific injury in this precise context (9). We need to go beyond traditional psychological models of sports injury. A qualitative method appears to be the most appropriate to explore this complex phenomenon and provide a contextual perspective of the problem, yielding insights not previously studied (9). Since biomechanical and physiological risk factors have already been widely reported, this study sought to determine the psychological and health-related risk factors for sustaining a first HSI in male competitive soccer players, considering them at personal, contextual and situational levels (17, 18).

## 2 Method

### 2.1 Participants

This study obtained approval from the local Ethics Committee for Non-Interventional Research (CERNI n°2021-05, presided by the Pr. Yves Strickler). Participants (*N* = 10, *M*_age _= 19.8 ± 2.6 years) were recruited via the authors' networks and staff of their clubs. Were included male competitive soccer players over 16 years old, French speakers, volunteers to participate. They should specify a minimum of 5 years of practice and train at least 3 sessions per week at the time of their first HSI (Table 1). On average, they had sustained their first HSI 1.9 ± 2.1 years before the interview. Participants with diagnosed psychiatric pathology, non-soccer-related hamstring injury, or incomplete interviews were excluded.

**Table 1:** Population characteristics at the time of the first hamstring strain injury.

Characteristics of the 10 participants at the time of their first HSI	Number or mean ± SD [range]
Age (years)	17.9 ± 1.8 [15–20]
Soccer experience (years)	11.0 ± 1.9 [9–15]
Training and match sessions/week	7.4 ± 2.6 [3–11]
Level	
Reserve of pro team	4
National (young)	3
Regional	2
District	1
Position	
Lateral	5
Midfielder	4
Defender	1
Living environment	
Parents’ house	5
Internship	2
Individual apartment	3
Main activity	
Student	6
Professional soccer player	4
Paid for playing	7

### 2.2 Procedure

We conducted a qualitative study using in-depth interviews and a thematic analysis approach. Criteria of rigor were ensured. The semi-structured interview guide was based on Meeuwisse's and Bahr and Krosshaug's multifactorial models of sports injury causation (17, 18), enriched from the literature on psychological risk factors in sports injury [e.g., the stress-injury model (10), biopsychosocial sports injury risk profile (19), Slimani et al.'s meta-analysis (16), and Teillol et al.'s qualitative study (14)]. Questions were semi-open-ended to allow new insights to emerge from the interviews. The guide covered soccer players’ individual psychological risk factors before the first HSI (e.g., psychological traits, health behaviors), context before the HSI, and situational factors at the moment of the injury. After a pilot interview (not included in our final sample), some questions were reformulated to enhance clarity. This final guide was used for all the individual interviews in our study (Supplementary File 1). For replicability, the first author, experienced in physiotherapy, conducted all interviews following academic semi-structured protocols. Interviews were conducted and recorded from May to October 2021 via secure Zoom (Mean interview time = 57 ± 7 min). They were transcribed verbatim before analysis. Data saturation was reached after analyzing the initial eight interviews, with no new themes emerging in the last two interviews. Consequently, we conducted ten interviews, aligning with recommendations in the literature to achieve saturation in a homogeneous population [9–17 interviews] (20). Further details are available in Supplementary File 1.

### 2.3 Analysis

To analyze the data, the authors used thematic analysis according to Braun and Clarke's methodology (21, 22), with both deductive (i.e., theory-driven) and inductive (i.e., data-driven) approaches, supported by Nvivo software (V1.5.2). The themes were based on the three main categories of Meeuwisse's and Bahr and Krosshaug's multifactorial models of sports injury causation (17, 18): individual, contextual and situational risk factors. To ensure coding reliability, the first and last authors independently coded three transcripts and reached consensus on their codes, based on the theory-driven subthemes explored in the interview guide. Subsequently, they collectively coded the following seven interviews. Employing an inductive approach, they identified new subcategories (i.e., not initially considered during the first coding), such as controlling coaching style, engagement despite pain, and health literacy. Lastly, the other authors reviewed the themes as disinterested peers. Using a mind map, they reached consensus about the number and labels of the subthemes.

## 3 Results

**Figure 1:**
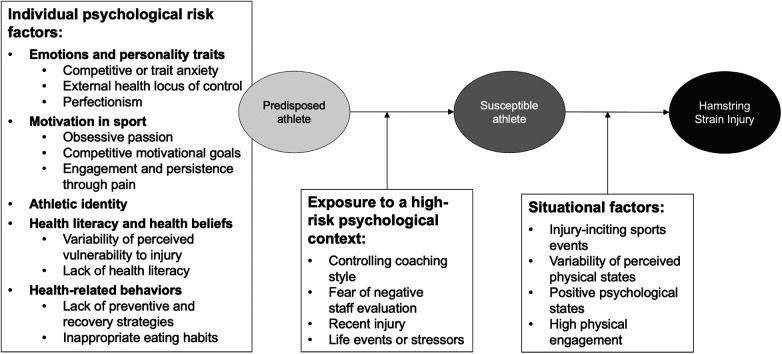
Comprehensive model of psychological risk factors for a first HSI in soccer adapted from Bahr and Krosshaug (18).

The researchers identified 452 statements classified into 13 subcategories and the three overarching themes of the interview guide (Figure 1). Each theme is presented below with its quantitative representation in our sample. We illustrated them in Supplementary File 2 with a representative quotation from the participants’ interviews. Participants are cited in the text, referred to by their anonymous code (p#n, *n* = 1–10).

### 3.1 Individual psychological risk factors

#### 3.1.1 Emotion and personality traits

While participants did not necessarily consider themselves as anxious, the majority of them exhibited signs of anxiety (80%). Some presented both somatic and cognitive anxiety in and out of competition, like p#10, whereas others described anxiety consequences (p#5). To make decisions about their health, 70% of the participants seemed to completely rely on staff (e.g., doctor, coach). They scrupulously followed staff directives, taking them literally (p#6), and did not spontaneously initiate health behaviors, like visiting the recovery room or skipping training due to a physical issue (p#2). This external health locus of control was associated with a very conscientious personality in our sample, with 70% showing perfectionism. They expressed perfectionistic striving and perfectionistic concerns, such as p#10. They were totally engaged in their activity, doing their best in every session and expressing fear of failure (p#1) that could reach all-or-nothing extremes (p#5).

#### 3.1.2 Motivation in sport

All the soccer players of our sample were very much intrinsically motivated to play soccer. Fifty percent of them spontaneously presented soccer as their passion (p#1) and the other 50% described how much they loved to play, talking about how important soccer is in their lives [e.g., “*It's above everything.”* (p#8), or “*second place after family*” (p#2, p#3, p#10)]. Even during their free time, they engaged in peer-led play and indirect involvement (e.g., watched soccer on TV, played soccer video games) (p#4). This obsessive passion meant that soccer came first at the expense of other areas of their lives (p#1). This was associated with social exclusivity, with most of their acquaintances involved in the soccer world (e.g., teammates, coaches) (p#3). This over-commitment to soccer practice and these social and educational sacrifices were driven by competitive motivational goals for 90% of the sample: signing a professional contract, being spotted for a professional team, or joining their club's top team (p#2, p#4). For 80% of the participants, these competitive goals were stronger than their health concerns, so they persisted in playing despite pain or unresolved injuries (p#4).

#### 3.1.3 Athletic identity

All participants displayed a strong athletic identity, describing several aspects of their soccer self-identity, their exclusivity around soccer, their soccer social identity, and their positive and negative affects experienced during play. (p#10). This athletic identity could be overly strong, as with p#1, who explained: “*I don't see myself doing anything else in life.”*

#### 3.1.4 Health beliefs and health literacy

Analyses reveal various perceptions of injury vulnerability in our sample. Despite prior injuries, 40% viewed themselves as not or minimally vulnerable (e.g., p#3: “*For me, there was really a 0% chance that I would get injured”)*, while 30% perceived themselves as very vulnerable (e.g., p#10: “I was too afraid of getting hurt.”). We also noticed that 80% of the soccer players in our sample presented a lack of knowledge about health-related topics (p#8). They didn't have sufficient knowledge about appropriate recovery strategies, good sleep management, proper food intake, or the most common injuries in their sport (p#7). The participants adopted passive informational strategies, waiting for the coach (30%) or the doctor (80%) to provide information or instructions (p#3). Otherwise, they followed their intuition (40%).

#### 3.1.5 Health-related behaviors

Possibly linked to low health literacy, participants showed little in the way of positive health-related behaviors. Despite being in a training program, 80% reported never or only occasionally performing specific exercises for HSI prevention, with half expressing skepticism about their effectiveness (p#2). Also, almost all the participants (90%) lacked recovery strategies even though they were encouraged to employ them with free access to dedicated facilities (e.g., stretching room, cold bath). After an important game or intensive training, they used only rest and sleep as strategies to recover (p#1, p#3). Sixty percent of participants had inadequate diets for their sports activity. Half did not eat fruits or vegetables daily, and despite training in the late morning, many skipped breakfast or morning meals. (p#1).

### 3.2 Exposure to a high-risk psychological context

In our sample, athletes presented risk factors predisposing them to injury and were exposed to a psychological context that could increase their risk of HSI, making them more susceptible to injury.

#### 3.2.1 Controlling coaching style

Ninety percent of the players described their coaches as employing a controlling coaching style and a top-down management within their club. Based on players' fitness questionnaires, staff determined playing time, training methods, and participation in “care sessions”. They also made health-related decisions, choosing return-to-play timelines after injuries, regulating sleep behaviors during live-in internships (e.g., curfews, smartphone use restrictions at night), and controlling body weight and eating schedules during the day. (p#3).

#### 3.2.2 Fear of negative staff evaluation

Throughout the interviews, a strong social desirability bias toward staff was observed. Fifty percent of the players aimed to impress the staff by demonstrating they were the best: highly involved, focused, reliable, and strong (p#3, p#7). This priority sometimes induced anxiety (p#2), with a fear of negative staff evaluation. Consequently, participants consciously concealed certain feelings or perceptions to avoid appearing weak or risking not playing (p#7).

#### 3.2.3 Recent injury

Every player had experienced at least one injury, and 60% had been injured in the months preceding their first HSI. Four participants indicated that they still occasionally use an orthotic device at the time of their HSI due to incomplete recovery from previous injuries (p#10).

#### 3.2.4 Life events or stressors

In the two months before their HSI, 80% of the participants had experienced difficult life events or life stressors (e.g., parents' divorce, romantic breakup, failed exams) (p#9, p#2).

### 3.3 Situational factors

Lastly, our susceptible athletes suffered their first HSI after they met situational factors.

#### 3.3.1 Injury-inciting sports events

Seventy percent of the HSI occurred during matches [in 60% of the cases while sprinting (p#9)]. Otherwise, they occurred during a high (or increasing) training load period (p#6, p#2). They got injured at the beginning of the season, during the championship restart (i.e., autumn; p#3; 40%); or in the middle of the season, when they played consecutive games (i.e., winter; p#7; 60%).

#### 3.3.2 Variability of perceived physical states

Participants reported heterogeneous perceptions of their physical states during the triggering event. Twenty percent perceived themselves as very vulnerable to injuries at this moment; they had pain (40%) or felt tired (60%) but wanted to play (p#5). On the other hand, 30% of our sample felt very well, neither in pain (60%) nor tired (40%) and did not perceive themselves as especially vulnerable to injury at this moment (p#10). Whether they perceived themselves as physically vulnerable or not at the time of the injury, they encountered a situation that made them more susceptible to adopt risky behaviors.

#### 3.3.3 Positive psychological states

Although a portion of our sample was not in an optimal physical state, all participants reported positive psychological states at the time of their injury (p#9).

#### 3.3.4 High physical engagement

Finally, 70% of the participants were highly physically engaged when they were injured. Two scenarios emerged: (a) participants experienced positive states (e.g., flow, pain-free) and played at a higher intensity than usual (p#3), or (b) players encountered an opportunity, like a decisive pass or a key shot, and “*gave it* (their) *all*” (p#5).

## 4 Discussion

Using Meeuwisse's (17) and Bahr and Krosshaug's (18) comprehensive models of injury causation, this study aimed to identify the psychological risk factors for sustaining a first HSI among male soccer players. They were categorized into three themes: individual, contextual, and situational risk factors (Figure 1).

The study confirmed that the individual-level risk factors for HSI align with those reported in the sports injury literature (10–12, 14, 16, 19, 23, 24). However, it also highlighted specificities of the soccer context for HSI beyond the factors of the stress-injury model (10). We observed an obsessive passion for soccer, an external health locus of control, and a lack of health literacy. These findings can be explained by the high media attention given to soccer in France and the common aspiration among young players to become professional. Soccer can easily become an obsessive passion, leading players to develop competitive motivational goals, perfectionism, and persistence through pain to cope with intense competition and gain the attention of recruiters. In some cases, this led them to hide injuries from staff. All these individual factors for sports injury (10, 11, 23) favor the development of anxiety and could be identified as risk factors for HSI.

The soccer academies and clubs studied were well-organized and encouraged players to consult staff for any health concerns. Nonetheless, this strong social support might unintentionally foster an external health locus of control and a lack of health literacy, as players did not seek out information independently. This might explain their lack of strategies to prevent injuries (24, 25), inappropriate eating habits, and lack of recovery strategies, also identified as risk factors for HSI. These factors, in line with the biopsychosocial sports injury risk profile (19) negatively impact the hamstring's ability to deal with high intensity demands (19, 26, 27).

Players of the study were in a context that potentially increased their susceptibility to injury. Before being injured, 80% of them had recently encountered life events or life stressors. This confirmed the importance of this psychological risk factor for sports (10, 11) and soccer (16) injuries, and also specifically for HSI. Previous injuries, already identified as a risk factor for HSI (6) and sports injury (10, 11, 18, 19), were also confirmed in this study. In line with previous research (12), the fear of negative staff evaluation was identified as a potential contextual risk factor for HSI in soccer. While HSI is typically considered as an acute injury, soccer players in our sample exhibited risk profiles similar to those of athletes experiencing overuse injuries (e.g., perfectionistic personality, strong athletic identity, persistence through pain, sense of duty toward the coach). The study identified a controlling coaching style as a specific risk factor for HSI. This top-down management approach restricted players' control over their own health and careers, potentially causing frustration, anxiety, and fear of failure (28), characteristics also observed in our sample. These contextual factors may be linked to players' high expectations and continuous staff evaluation, exacerbated by competitive goals, anxiety, and perfectionism, all predisposing players to HSI.

The players, already susceptible to HSI, were injured when they encountered situational factors that acted as a trigger. Consistent with the literature, most HSIs occurred during matches (1, 6, 7, 29), at the beginning of the season or in winter (29) and when players were sprinting (1, 29), or highly physically engaged (14). In these situations, players often exceeded their limits, exposing themselves to extreme physical demands that led to HSIs (14). Outside of matches, increased training load was identified as a potential risk factor for a first HSI, aligning with the literature (7). It induced temporary physical and psychological changes that could affect players' perceived vulnerability to injury. Despite feeling vulnerable, some players continued to play, ignoring pain or fatigue, until being injured. This mechanism, already identified by Teillol et al. (14), seems to be a risk factor for HSI. Other players, however, felt quite well, invincible even, at the moment of injury and played with greater intensity than usual, increasing the physical load and putting them at risk (26). These psychological factors can collectively contribute to a loss of vigilance, which is a common trigger for sports injuries (10).

### 4.1 Limitations and future research

Despite its rigorous methodology, this study has limitations. Interviewing participants about past events might introduce recording bias. Also, these results are exploratory and cannot be generalized to women or to other sports. Prospective quantitative studies are needed to validate these findings among larger samples. Future research could examine whether interventions targeting the psychological risk factors identified in this study—for example, promoting player autonomy by moderating controlling coaching styles and enhancing health literacy—could improve compliance with well-established preventive programs, such as the Nordic hamstring exercise (25, 27, 30) and reduce the HSI rate in soccer clubs. Finally, it is crucial to investigate the recursive pathway adaptations for susceptible athletes (31), and psychological risk factors associated with HSI reinjury.

### 4.2 Practical implications

From an applied perspective, clubs should aim to identify psychological risk factors associated with the first occurrence of HSI within their team. Improving health education and promoting player autonomy can enhance internal health control, raise pain awareness, encourage healthier behaviors, and mitigate the fear of staff evaluation. Offering regular psychological support through private sessions should help players handle life challenges without stigmatization. Clubs should adopt a comprehensive approach that addresses both individual and contextual risks. More suggestions are available in Supplementary File 3.

## 5 Conclusion

This study identifies psychological risk factors for soccer-related HSI based on injury causation models (17, 18). It extends the stress-injury model determinants (10) with biopsychosocial and contextual variables (9, 11). It revealed that predisposed players lacked health literacy, ignoring physical and psychological consequences of persisting through pain, overexertion, life events, or prior injuries. Driven by perfectionism, competitive motivational goals, and fear of negative evaluations, they exceeded their limits, seizing sudden opportunities during matches. Thus, staff should preemptively identify those individual and contextual HSI risk factors among their players. Particularly, the results suggest addressing controlling coaching style, improving health literacy, and fostering player autonomy to improve HSI prevention. This would mitigate the negative consequences of perfectionism, the fear of negative staff evaluation, and the adoption of risky situational behaviors. It would also increase the adoption of preventive measures by making players aware of the risks associated with their behaviors and soccer practice.

## Data Availability

The datasets presented in this article are not readily available because all data relevant to the study are included in the article or uploaded as online supplemental information. Due to the sensitive nature of the research and French laws protecting the privacy of health-related information, quotations can be provided but full transcribed interviews are not available. Requests to access the datasets should be directed to stephanie.meriaux@univ-cotedazur.fr.
