# Supplementary material for: Psychological risk factors for a first hamstring strain injury in soccer: a qualitative study
Source: Front Sports Act Living. 2024 Jun 14;6:1377045. doi: 10.3389/fspor.2024.1377045 (PMC11211564; doi:10.3389/fspor.2024.1377045)
Supplement: Supplementary file 1 [file Datasheet1.pdf]

Supplemental File 1: Interview guide

Interview guide

Objective of the study: To characterize HSI experience of soccer players and identify their primary risk factors to sustain a HSI at the individual, contextual and situational levels.

Population: The participants were competitive male soccer players, over 16 years old, fluent French speakers, training at least three times per week at the time of their first HSI, experts in the activity (at least four years of competitive soccer experience when they get injured), and still practicing soccer.

Non-inclusion criteria: Psychiatric or cognitive deficiencies; first HSI more than 6 years ago; return to sport less than 3 months ago, athletes injured at the time of the interview (other than HSI).

Recruitment procedure: First, recruitment posters were distributed via social networks and contacts in soccer clubs. The first author personally contacted potential soccer players who expressed interest in participating in the study. During these phone calls, she provided detailed information about the study's objective and procedures and confirmed that the participants met the inclusion criteria. Prior to the interview, a follow-up email was sent to each participant containing a recapitulative information notice and a consent form. All participants were required to provide their informed consent to participate in the study before the interview began.

1 Introduction of the interview

- The meeting took place in an online video conference via a secured Zoom program.
- Participant and interviewer were in a quiet and isolated setting to be able to speak freely.
- The interviewer recalled the objective of the study and the way the interview would be conducted.
- The interviewer asked if the participant had any questions, answered them and checked the signature on the consent form.
- The interviewer reminded the participant of the confidentiality of the collected data.
- Anonymization was ensured by using status instead of name for the stakeholders (e.g., trainers, doctors, friends) and not naming clubs or cities.
- The recording agreement was checked one last time before starting the interview.
- Before recording, general information about the participants was completed (e.g., experience in the sport, date of the first HSI, living place, position, compensation for playing).

2 Interview guide

Structure of the interview guide with examples of questions.

2.1 Individual psychological factors

"I'm now going to ask you some questions about your personal characteristics and psychological profile before you had your first hamstring injury."

2.1.1 General motivation for soccer

- Why did you practice soccer? [Can you elaborate? What did you enjoy about this sport?]

- What were your other hobbies?

- What made you succeed in obtaining this playing level?

2.1.2 Emotions and general personality

- In general, how would you be described? [Why or why not? Could you give me an example?]

- How did you feel going into competitions? [What was your mindset?]

- These emotions that you describe, did you usually feel them outside of competition periods? [Can you be more specific?]

2.1.3 Perceived vulnerability to injury

- Had you seen another player suffer a hamstring injury (or other serious injury) in your sport before your HSI? What did you think about it? [Please elaborate.]

- How much did you feel you were at risk of injury?

-What were you doing to limit the injury risk? [Please elaborate.]

2.1.4 Health-related behaviors

- When you were tired or after an intense event (physically and/or mentally), how did you recover? What other recovery strategies did you use? Why did you do it this way? How did you learn about these strategies?

- In general, how did you get information about your health, sport or training?

- How would you describe your health lifestyle at the time? Why?

- How did you manage your sleep? What about the night before the games?

- How did you prepare your meals? Can you please describe your diet on a typical weekday? What were the changes at the weekends?

- What was your daily water intake? What other drinks did you consume?

2.1.5 Reformulation of the elements of this part by the interviewer

2.2 Psychological contextual factors

"I would now like us to put ourselves in <u>the context of your first hamstring injury</u>."

2.2.1 Training load

- How was your training load managed?

- How did you adapt to cope with heavy training loads? Did you have planned relief or rest periods in your training?

2.2.2 Social support

- What was the atmosphere in the team? What was your relationship with the other players? And the staff?

- What did the people around you think about your sport? How did they react?

- What was your relationship with your coach? [Please explain. What were their expectations? How did this show up?]

- What did you say to each other when you disagreed? What did you do?

2.2.3 Life events or stressors

- How did you manage your personal and professional activities to fit everything together?

- Had anything changed in the weeks before your injury or any significant life events? [Please elaborate.]

- Did you have any other injuries before your first HSI? Which ones?

2.2.4 Reformulation of the elements of this part by the interviewer

2.3 Situational factors

2.3.1 Injury occurrence and context

- Could you describe to me how your injury occurred?

- What was the context for it?

- What was your mindset (thoughts - emotions) when you went to training/match just before the injury? [Why?]

- Physically, how did you feel before this practice/game?

2.3.2 Injury diagnosis

- Can you tell me which leg was injured and what grade of injury was diagnosed?

- By whom and how soon after your injury was the injury diagnosis made? How was it done?

2.3.3 Reformulation of the elements of this part by the interviewer

2.4 Free speech

"We've arrived at the end this interview, is there anything else you'd like to share?"

2.5 Conclusion and acknowledgments
