# Supplementary material for: Psychological risk factors for a first hamstring strain injury in soccer: a qualitative study
Source: Front Sports Act Living. 2024 Jun 14;6:1377045. doi: 10.3389/fspor.2024.1377045 (PMC11211564; doi:10.3389/fspor.2024.1377045)
Supplement: Supplementary file 2 [file Datasheet2.pdf]

Supplemental File 2: Table of quotations

Emerging risk factors	Frequency	Code	Supporting quote
**Individual psychological risk factors**			
Emotions and personality traits			
Competitive or trait anxiety	80%	p#10	p#10: *"I had sweaty hands, a stomachache, and trouble falling asleep the night before a game." […]* Interviewer: *"Is this something you used to feel outside of competition periods?"* p#10: *"Yes. I get stressed a lot, for anything."*
		p#5	*"Asking myself questions often made it hard to fall asleep [...] I felt a lot of tension as well."*
		p#1	Interviewer: *"In general, how did people describe you?"* p#1: *[…] "Sometimes, a stressed-out person"*
External health locus of control	70%	p#2	*"When I wasn't in care, on the schedule, well I didn't go in by myself."*
		p#6	*"I can disagree, but I can't prove him wrong, because he's my coach. So I listened to what he told me and I thought it was the best solution for me."*
		p#7	Interviewer*: "When you wonder about something, how do you usually go about getting answers to those questions?"* p#7*: "You go to the doctor and he'll tell you, you ask him for advice."*
Perfectionist personality	70%	p#10	• *"The biggest goal was my dream, to be a professional player."*<br>• *"I told myself that I had to do better. I was always like that."*<br>• *"I used to get upset about the things I did wrong."*
		p#1	*"I can't have a bad game, I have to play a good game, I have to keep performing well so I can keep hoping to have something."*
		p#5	*"I do things, I do them thoroughly, otherwise I don't, I'm not interested."*
Motivation in sport			
Obsessive passion	100%	p#1	"(I like) *Everything* (in soccer)*! I like to run, to surpass myself and to win matches. I don't know, it's a passion. It's […] like a drug for me. I like it so much."*
		p#4	"*I am a passionate person, I live soccer, I eat soccer, I watch soccer, that's all I do, and that's all I like.*"
		p#1	*"Soccer comes first. Whether it's my buddies and I can go out with them and I have a match the next day, well I don't go out."*
		p#3	*"I had a breakup with a girlfriend of a year and a half. [...] she was the only person I could maybe trust outside of boarding school [...] I don't have any other friends [...] who are not from boarding school."*

Competitive motivational goals	90%	p#2<br>p#4	"(My goal) *was to play in the national U17 team and to become a professional soccer player."*<br>*"My objective was to play in the pros group. To perform well. That's it."*
Engagement and persistence through pain	80%	p#4	*"I had a little bit of pain in my testing, but I wanted to keep playing a little bit, I mean, I thought I could push it. I pushed it 2, 3 days, and after the third day I stopped, […] I went to do my ultrasound, and I have a grade 2."*
Athletic identity	100%	p#10	• *"My dream had always been to be a professional player."*<br>• *"It was up, soccer, sleep, soccer: all soccer!"*<br>• *"They* (his parents) *have pushed me since I was a little kid to do that. My dad [...] was always behind me [...] He came to all the games."*<br>• *"As long as I'm playing soccer, I'm happy."*<br>• "(When I did not perform well,) *[…] I was angry, frustrated."*
		p#1	*"I don't see myself doing anything else in life."*
Health literacy and health beliefs			
Low perceived vulnerability to injury	40%	p#3	*"For me, there was really a 0% chance that I would get injured. When it happened, I was really surprised."*
High perceived vulnerability to injury	30%	p#10	*"I was afraid it would happen to me too. So I was more careful about what I did on the field […] I was too afraid of getting hurt."*
Lack of health literacy	80%	p#8	*"I mean, I didn't know […] how to get rid of this feeling of tired legs, all that. […] I didn't have any advice, we'll say, so I was doing it the way it felt right."*
		p#7	*"I didn't even know what it was* (an HSI), *me, at first."*
		p#3	*"I get information... With information that is generally given on the field."*
Health-related behaviors			
Lack of preventive and recovery strategies	90%	p#2	*"Before, it's true that I didn't think it* (preventive exercises) *worked very much. I didn't do much of it either."*
		p#3	*"Before my first injury, I was just thinking about sleeping. I wasn't thinking about stretching, I wasn't thinking about recovering with cold baths or anything."*
		p#1	*"I don't know, it (recovery) happened."*
Inappropriate eating habits	60%	p#1	*"In the morning, at that time, I didn't eat. I didn't eat, I only ate at noon and in the evening. And I had pasta, rice at every meal. There was no vegetable, no fruit."*

Exposure to a high risk psychological context			
Controlling coaching style	90%	p#4	• *"Before the training session, he* (the doctor) *calls us, and he does a little testing to see if we can train or not."*<br>• *"He always wins at the end, because he's the coach."*
		p#3	"We *don't really have the right to do this* (take snacks)*"*.
Fear of negative staff evaluation	50%	p#3	*"Here you have to be serious, you are judged on a daily basis. You have to work on a daily basis too."*
		p#7	*"On the field, I was showing him (the coach) that I was there, that I was present."*
		p#2	*"I was feeling stressed, a little under pressure… to know if I was going to be taken into the group."*
		p#7	*p#7: "If you're tired or whatever, if you have aches or pains or whatever, you have to tell the physical trainer, so he can let you rest or whatever. And even from yourself, you feel it." Interviewer: "Do you say it easily, you, if there are... things?" p#7: "No, not really. No, not really. [...] I prefer to be on the field."*
Recent injury	60%	p#10	*"After that injury I always played with my ankle strapped."*
Life events or stressors	80%	p#9	*"I had a competitive examination [...]. And so, I couldn't go because there was a train problem, I arrived late and I couldn't take the exam. [...] It was a total failure. [...] And I only have the right to one try."*
		p#2	*"My parents got divorced maybe uh... a month, two months... two months before."*

Situational factors			
Injury-inciting sports events			
Beginning of the season	40%	p#3	*"It was after the third championship game."*
Middle of the season	60%	p#7	*"It was in the middle of the season."*
Heavy training load	30%	p#6	*"No* (nothing has changed before my HSI), *just, I'm thinking about the work overload […] I was coming back from injury and I couldn't handle all the sessions."*
		p#2	*"At that time, [...] we were increasing the training load."*
Match<br>Sprint	70%<br>60%	p#9	*"It was an away game. It was raining heavily and I ran a sprint. And then I fell down, I couldn't get up, it hurt, it got pulled. I knew it had broken somewhere."*
Variability of perceived physical states			
Experienced negative perceptions of their physical state	60%	p#5	• *"Physically, I was fine, a little worn out, yeah, a little worn out, tired, heavy legs, broken, stuff, I had, well, I had my hamstring pulling a little bit."*<br>• "(I felt) *100% (at risk of injury). It's true, it's the feeling. Anyway, […] it's the desire to play that takes over."*<br>• *"Since I was in pain, I was managing the effort I was putting in."*
Experienced positive perceptions of their physical state	70%	p#10	Interviewer: *"Physically, how did you feel before that game?"*<br>p#10: *"Very good because it's also the year, well, I'm having my biggest year. So yeah, very, very good."*
Positive psychological states	100%	p#9	*"I was too happy to go because it had been 2 weeks since I had been there."*
High physical engagement	70%	p#3	*"I was so happy to feel nothing, to feel no pain.[…] I played my heart out […] I felt invincible."*
		p#5	*"This was a golden opportunity! [...] It was at that moment that I accelerated and I said to myself: 'I'm going,' and when I went, there you go. […] I gave it my all. I gave a little too much."*
