# Supplementary material for: Psychological risk factors for a first hamstring strain injury in soccer: a qualitative study
Source: Front Sports Act Living. 2024 Jun 14;6:1377045. doi: 10.3389/fspor.2024.1377045 (PMC11211564; doi:10.3389/fspor.2024.1377045)
Supplement: Supplementary file 3 [file Datasheet3.pdf]

Supplemental File 3: Recommendations

What can be changed to mitigate the psychological risk factors of HSI in soccer?

- **Promote players' autonomy** by pointing out the purpose of staff demands and the possibilities for adapting their programs and participating in their health and training decisions. This would decrease the perception of a controlling coaching style and make them actors in their health and careers.

- **Provide health education to players**, notably through health-literacy development (e.g., nutrition, prevention, recovery, injury management).

- **Encourage the players to extend their social networks** (e.g., sports social networks and school or professional social networks) **and practice other sports and leisure activities**. This would avoid early sports specialization, moderate their athletic identities, and reestablish a harmonious passion for soccer.

- **Adapt the training load to stressors and life events.** This would avoid over-solicitations at a time when the players are experiencing psychological issues.

- **Systematize the psychological follow-up of the players**. This would provide support without the risk of stigmatization since everyone on the team would have regular appointments. It would help them to deal with emotions about life events, fears of negative staff evaluation, return to play after injury, anxiety, perfectionism, or obsessive passion.

- **Promote mastery achievement goals**, by giving the players the right to make mistakes without consequences, such as the team composition for the next match. This would decrease the fear of failure and encourage them to pay attention to pain and report it to the staff, preventing injuries from getting more serious and negatively impacting individual careers and team performance.
